# Quality of life and understanding of disease status among cancer patients of different ethnic origin

**DOI:** 10.1038/sj.bjc.6601159

**Published:** 2003-08-12

**Authors:** N Tchen, P Bedard, Q-L Yi, M Klein, D Cella, S Eremenco, I F Tannock

**Affiliations:** 1 Princess Margaret Hospital and University of Toronto, 610 University Avenue, Toronto, Canada M5G 2M9; 2 Center on Outcomes, Research and Education (CORE) Evanston Northwestern Healthcare 1001 University Place, Suite 100, Evanston, IL 60201, USA

**Keywords:** quality of life, ethnic origin, primary language, language of consultation, understanding of disease status

## Abstract

Patients managed in European or North American cancer centres have a variety of ethnic backgrounds and primary languages. To gain insight into the impact of ethnic origin, we have investigated understanding of disease status and quality of life (QoL) for 202 patients. Patients completed questionnaires in their first language (52 English, 50 Chinese, 50 Italian, 50 Spanish or Portuguese), including the Functional Assessment of Cancer Therapy – General (FACT-G) QoL instrument, questions about disease status, expectations of cure and the language and/or type of interpretation used at initial consultation. Physicians also evaluated their status of disease and expectation of cure, and performance status was estimated by a trained health professional. The initial consultation was usually provided in English (except for 32% of Chinese-speaking patients); interpretation was provided by a family member for 34% of patients with limited English proficiency (LEP) and by a bilingual member of staff for 21%. Patients underestimated their extent of disease and overestimated their probability of cure (*P*=0.001 and <0.0001, respectively). Estimates of probability of cure by the English speakers were closer to those of their physicians than the other groups (*P*=0.02). English-speaking patients reported better and Italian-speaking patients poorer overall QoL (*P*<0.001 for Italian *vs* other groups). Performance status was correlated with QoL and most closely related with the extent of disease. Understanding of cultural differences is important for optimal management of patients with cancer.

In many countries and for many ethnic groups, disclosure of the diagnosis of cancer to patients, as well as information regarding their stage of disease and prognosis, remain controversial issues in the doctor–patient relationship. A barrier to doctor–patient communication is also introduced when patients do not speak the same first language as their physicians, as is common for patients from some immigrant populations in North America and Europe. Often, there are substantial differences between the information that the physician has tried to convey, and that understood by cancer patients (Olweny, 1997). The quality of information understood by patients may be important for their understanding of the evolution of their disease and their compliance with treatment regimens. Poor understanding because of difficulties of language and interpretation might lead to suboptimal management of cancer patients.

Disclosure of information about serious illness varies markedly among different countries and ethnic groups due to differing cultural, ethical and legal concerns. In general, North American and Northern European populations expect more complete disclosure of information than Southern European populations (Perez-Stable *et al*, 1992; Mitchell, 1998; Galanti, 2000), although a survey of Portuguese-speaking cancer patients found that most of them desired full information (Pimentel *et al*, 1999). A survey comparing perceptions of cancer by people of Southern European origin (Latinos) and English speakers (Anglos) found that Latinos were more likely than Anglos to believe that having cancer is like getting a death sentence, and that they would prefer not to know if they had incurable cancer (Perez-Stable *et al*, 1992). Beliefs about cancer also differ among ethnic groups: for example, some Hispanic patients believe that ‘its a punishment of God’ and/or that ‘destiny cannot be changed’ (Shankar and Figueroa-Valles, 1999). In some Asian countries, many patients do not know what cancer is (Phipps *et al*, 1999), or have major misconceptions about the disease (Bottorff *et al*, 1998).

The above studies suggest that the quality of life (QoL) of patients with cancer may depend not only on physical symptoms due to the disease and its treatment, but also on knowledge, or lack thereof, about the status of their disease. Several studies have analysed the relationship between characteristics of patients from different backgrounds and their disease and health-related QoL. Factors that contribute substantially to variations in QoL include ethnic/cultural origin (Kim and Rew, 1994; de Haes and Olschewski, 1998), as well as performance status, overall expectations of treatment, age, living conditions and the type of care that patients receive (Ganz *et al*, 1993; Wan *et al*, 1997). If different components of QoL have a variable impact on overall QoL among people speaking different languages and of different ethnic origin, it is important to develop questionnaires that are valid for different populations. The translation of questionnaires developed in one culture into the language of another can be problematic because of difficulties in achieving equivalent conceptual dimensions (Cella *et al*, 1993; Hays *et al*, 1993; Sartorius and Kuyken, 1994; The WHOQOL Group, 1994; Yabroff *et al*, 1996; Ware *et al*, 1998). Despite these problems, several QoL questionnaires, including the Functional Assessment of Cancer Therapy – General (FACT-G) instrument used in the present study, have been translated into many languages and validated for different ethnic groups (Aaronson *et al*, 1992; Bonomi *et al*, 1996).

Toronto is a multicultural city, with about 50% of the population of Caucasian origin who use English as their first language, while the remaining 50% includes several large (and many smaller) ethnic minorities with different cultures and languages. The problem of providing optimal care to patients of diverse ethnic origin is particularly common in Toronto, but reflects that of many other large North American and European cities. We therefore planned the present study to provide preliminary information about possible inter-relationships between the first language of patients, their perceptions of their disease and their QoL. We also sought to determine the possible influence of the language used in the initial consultation and the type of interpreter, if presented in English. All questionnaires, including the consent form, were presented in the first language of the patients. We included performance status, assessed by a health professional, because this might reflect aspects of QoL that are less dependent on language, ethnic origin and cultural beliefs. Our study was exploratory, but based on clinical observation and on literature cited above we established the following primary hypotheses.

1. English-speaking patients will have better knowledge and understanding of their disease than those who speak primarily Southern European or Chinese languages.
2. Patients with poorer knowledge of their disease status will have poorer QoL.

## PATIENTS AND METHODS

### Patients

This was a cross-sectional study in which patients were assessed once during a visit to a clinic at the Princess Margaret Hospital, Toronto, Canada's largest cancer centre. The study was approved by the Research Ethics Board of the Institution.

Patients were included in the study if they had a malignancy of any type or stage, and were being treated or assessed as outpatients. They were eligible if they were older than 18 years and belonged to one of the following language groups: English, Italian, Chinese (Mandarin or Cantonese, with a common written language) or Spanish/Portuguese. Patients with a history of psychiatric disease, drug or alcohol abuse or major chronic illness other than cancer were excluded. Eligible patients were provided with a consent form that had been professionally translated into their own language. Consenting patients then completed the questionnaires described in the following section.

### Questionnaires

Forms were constructed to assess demographic information, the patient's knowledge about the status of their disease and their expectation of cure and their satisfaction with the information received. The demographic questionnaire asked about age on leaving school and about further training or education; whether the patient was working and if so the type of work and income bracket; whether they were living with a partner and had children at home; whether they were born in Canada and if not at which age they immigrated; their first and second languages at home; and their religion (if any). The second questionnaire asked patients to rate their symptoms on a 0–4+ scale with verbal descriptors similar to those used to generate the ECOG Performance scale. It asked them to name their type of disease, whether it was a particular type, and which parts of the body were affected. It provided a check list of treatments received during the last 3 months and asked if doctors had taken time to explain the nature of disease and the chance that it might be cured (with yes/no answers). The form asked if the patients would like to know anything else (yes/no), with an open format to indicate what, and asked them to rate satisfaction on a 1+ (not at all satisfied) to 5+ (very satisfied) scale. It asked about language of initial consultation, and if English was used, whether there was translation and by whom. Finally, it asked patients ‘What do you think is the chance that your disease will be cured?’ and asked them to circle their best estimate (choices from 0 to 100% in 10% increments). Patients then completed the FACT-G QoL questionnaire followed by questions about whether they thought any items were missing or difficult to understand, and whether it was upsetting, indiscreet or annoying.

Forms generated for this study, including the consent form, were translated by professional translators into each of the four languages. Translations into each language were reviewed by focus groups of 3–4 bilingual volunteers, and their feedback was used to improve linguistic and conceptual equivalence. The translated questionnaires were then pilot-tested on 27 patients and final adjustments were made before proceeding with the study.

The FACT-G has been translated into 43 different languages using an established and rigorous translation methodology (Bonomi *et al*, 1996). The FACT-G is a 27-item compilation of general questions divided into four domains or subscales (Physical Well-being, Social-Family Well-being, Emotional Well-being and Functional Well-being). The total QoL score can be derived from the sum of the four subscale scores. In the present study, Version 4 of the FACT-G was used (Cella, 1997). Each patient completed the FACT-G in his/her first language.

One of the physician investigators reviewed the patient record and completed the ‘evaluation of disease by physician form’. If necessary he/she sought the advice of the managing physician to estimate the probability of cure of the disease. Performance status, using the Eastern Co-operative Oncology Group scale, was assessed by a trained research assistant (Oken *et al*, 1982).

### Statistical analysis

This study was exploratory in nature, and there were insufficient data in the literature to provide expected differences that relate to our primary hypotheses. The sample size for the present study was therefore based on feasibility rather than on expected differences in outcome. However, with the available sample size (52 English speakers and 150 non-English speakers), there is about 80% power to detect a 20% difference in the primary end points between speakers of different languages.

Descriptive statistics were used to analyse the demographic data for each language group, and the scores on the QoL questionnaire, its sub-scales and the global QoL score. Descriptive statistics were also used to describe the patient's knowledge of disease status, their estimates of likelihood of cure and their satisfaction with the information that they had received.

Disease status was classified as local, locoregional or metastatic (obtained from the hospital record), and agreement between patients and doctors with respect to this classification was compared using the McNemar *χ*^2^ test. The difference between expectations of cure by patients and doctors was compared using the paired *t*-test. The relationship between the accuracy of the patient's assessment of their disease status and probability of cure (as defined by physicians) and language of first consultation, type of interpretation, and ethnic origin were further examined using the *χ*^2^ test or ANOVA as appropriate.

To establish whether differences in language groups influence QoL scores, both univariate and multiple variable models were used to examine unadjusted and adjusted effects on QoL. In the multiple variable model, the demographic factors of gender, age, marital status and level of education, as well as status of disease were considered as potential confounders. A second multivariate analysis explored the effects of language group on performance status, with inclusion of the same potential confounding variables. The variables in the two models were designated prior to study analysis and not selected on the basis of a statistical rule; all of the potential confounding variables were kept in the model regardless of their significance in univariate analysis.

We used the Spearman correlation coefficient to determine possible relationships between (i) knowledge of disease stage (correct or incorrect assessment of local, locoregional or metastatic) and (ii) difference in expectation of cure between doctor and patient, with overall QoL, as determined by the score on the FACT-G. In a hypothesis-generating analysis, we used similar methodology to explore relationships between accuracy of knowledge of disease and probability of cure with the four domains of QoL defined by the FACT-G: physical, emotional, social/family and functional.

All data storage and analysis were performed using the Statistical Analysis System (SAS) software package (SAS Institute Inc, 1990). Corrections were not applied for multiple comparisons. Apart from tests of the *a priori* hypotheses, only *P*-values <0.01 should be regarded as suggestive of significance, and results should be regarded as exploratory.

## RESULTS

### Patients

A total of 202 patients were recruited: 52 English-speaking, 50 Chinese-speaking, 50 Italian-speaking and 50 Portuguese or Spanish-speaking patients. Of patients who were invited to participate in the study, 15% (nine out of 61) of English, 18% (11 out of 61) of Chinese, 25% (17 out of 67) of Portuguese or Spanish and 47% (44 out of 94) of Italian-speaking patients refused to participate (*P*<0.0001 Italians *vs* others).

Participants were first asked which language they spoke at home and whether they were born in Canada. Among the English-speaking group, 75% were born in Canada, but only 4% of the Italian-speaking, and none of the Chinese, Portuguese or Spanish-speaking participants were born in Canada.

The sample demographics of age, educational level, employment, annual income, living situation, performance status, type of cancer and disease status are shown in Table 1

**Table 1:** Sociodemographic characteristics of study sample

**First language**	**English (*n*=52)**	**Chinese (*n*=50)**	**Spanish/Port. (*n*=50)**	**Italian (*n*=50)**	***P*-value (overall)**
Median age (years)	55	52	55	64	*0.0001*
Interquartile range	46–64	44–59	47–68	56–72	
					
Education					
School only (%)	8	38	51	54	<*0.0001*
Further education (%)	43	34	34	44	
University educated (%)	49	28	15	2	
					
Employment (%)	38	28	32	10	*0.01*
					
Annual income					
(median, CAD$ × 1000)	30–40	20–30	20–30	20–30	*0.001*
					
Living with a partner (%)	67	84	75	72	*0.27*
					
Performance status					
0 (%)	43	31	26	32	*0.06*
1 (%)	47	38	39	32	
2/3 (%)	10	31	35	36	
					
Type of cancer					
Breast (%)	32	42	25	13	
Gynaecological (%)	12	7	8	9	
Head and neck (%)	2	31	2	0	
Gastrointestinal (%)	4	4	13	27	
Genitourinary (%)	18	0	17	9	
Haematologic (%)	18	4	13	20	
Lung (%)	2	7	2	7	
Other (%)	10	4	21	16	
					
Disease status					
Local (%)	23	30	25	27	*0.57*
Locoregional (%)	21	31	22	15	
Metastatic (%)	56	39	53	58	

. The median age was 56 years for the entire population, with older patients in the Italian patient group (median 64 years). Consistent with their older age distribution, fewer Italian-speaking patients were working (10% compared to 28–38% in the other groups). The English-speaking group had better Performance Status (*P*=0.004; English-speaking *vs* others). The overall differences in age distribution, education, employment and income were statistically significant. A major difference between the groups is that the Chinese-speaking population included a high proportion of patients with nasopharyngeal cancer, which is common in this ethnic group due to endemic infection with the Epstein–Barr virus.

### The initial consultation

**Figure 1:**
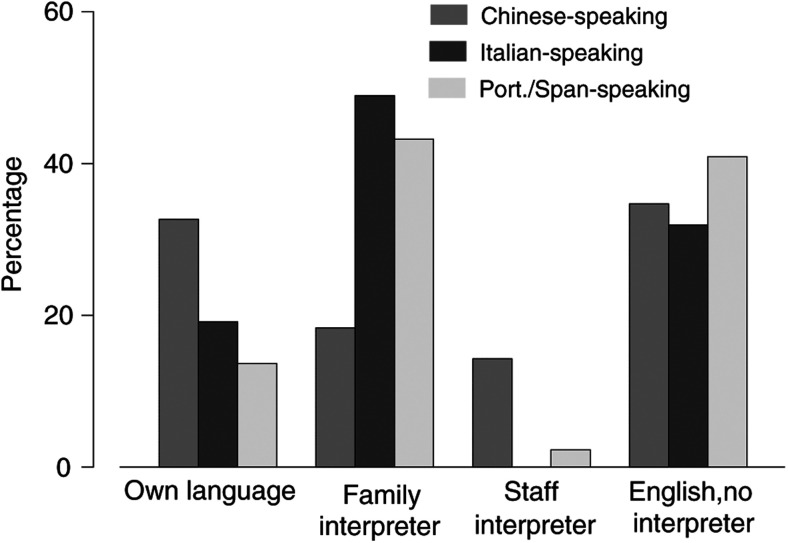
Language and type of interpretation at first consultation. (15 patients – 10 Chinese, three Italian and two Spanish/Portuguese-speaking – indicated a mixed consultation using their own language and English with interpretation and are represented in both categories in the figure.)

For about one-third of the patients in each ethnic group with limited English proficiency (LEP), the initial consultation was received in English without interpretation (35% for Chinese, 32% for Italian and 40% Spanish or Portuguese-speaking patients) (Figure 1

). Of the Chinese-speaking patients, 32% received an initial consultation in their own language from Chinese-speaking physicians. Interpretation was provided by a family member for 18% of the Chinese, 49% of the Italian and 43% of the Spanish or Portuguese-speaking patients. A total of 15 patients (10 Chinese, three Italian and two Spanish or Portuguese-speaking) indicated more than one option for the language of consultation and its interpretation, indicating the use of different strategies in different parts of the consultation.

**Figure 2:**
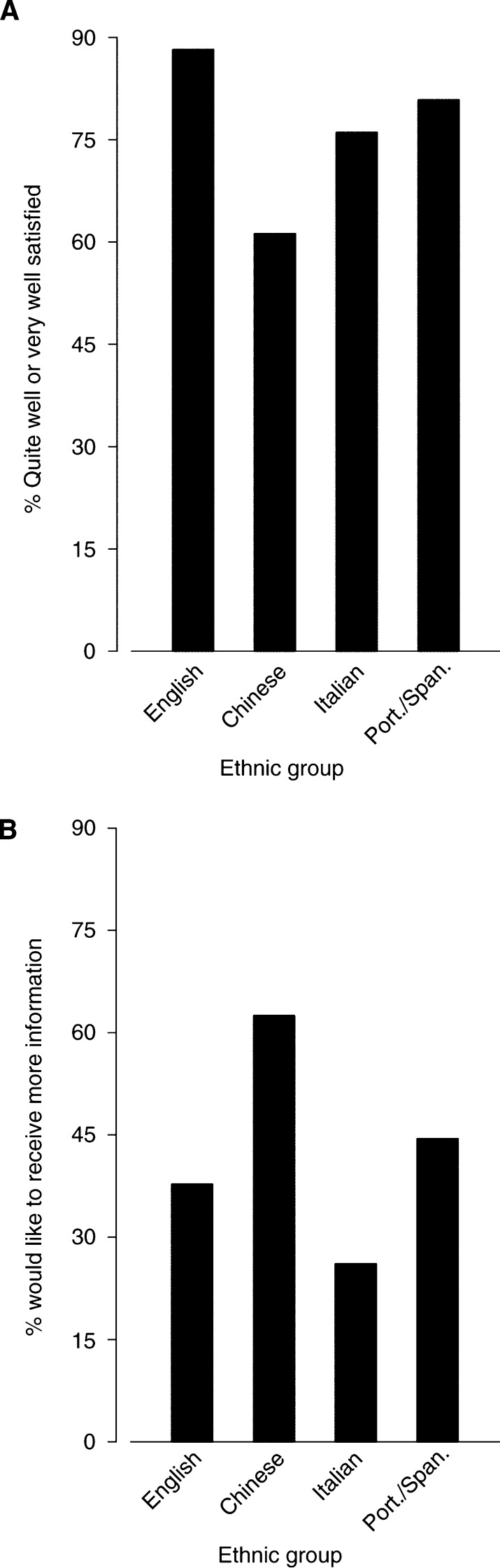
Satisfaction with the information received. (**A**) A Percentage of patients who are quite well or very well satisfied. (**B**) Percentage of patients who would like to have received more information.

Most of the patients were either ‘quite’ or ‘very’ well satisfied with the information received (Figure 2A

). However, many patients would like to have received more information about their disease: 38% of English, 63% of Chinese, 26% of Italian and 44% of Spanish or Portuguese-speaking patients (Figure 2B).

### Understanding of disease status and probability of cure

Some patients found these questions difficult and 56 (28%) of them did not provide information about the extent of their disease and 40 (20%) did not estimate their chance of cure. Differences between the responding patients in the four language groups regarding their understanding of the stage of their disease and that indicated by their hospital record are shown in Table 2

**Table 2:** Estimates of extent of disease by physicians and patients

		**Physician**		
		**Locoregional**	**Metastatic**	**Total**
Patients	Locoregional	45	43	88 (63%)
	Metastatic	17	35	52 (37%)
	Total	62 (44%)	78 (56%)	140 (100%)

*Note*: Data were not available for 62 patient/doctor pairs. McNemar's paired test *P*-value=0.001.

**Figure 3:**
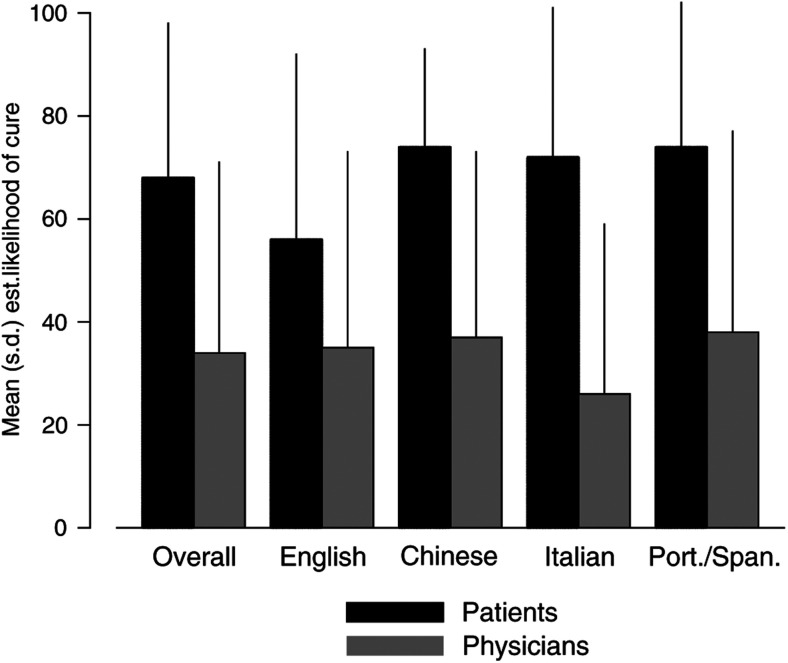
Estimates of likelihood of cure of their disease by patients in the four language groups in comparison to physician estimates.

; these differences are highly significant (*P*=0.001). More than half of the patients with metastatic cancer believed that their disease had not spread, although 27% of the patients with locoregional disease believed that they had distant metastases. Essentially, all patients estimated their probability of cure to be higher than that estimated by physicians (*P*<0.0001), and median values of these estimates are shown in Figure 3

. The difference between patient and physician estimates of probability of cure was greater for the LEP patients (means: 73 *vs* 33%) than for English-speaking patients (means: 56% *vs* 35%) (*P*=0.02 for difference of English *vs* LEP groups).

For the LEP groups, we analysed the influence of language of consultation and the type of interpretation used (if any) on (i) accuracy of the patient's understanding of disease status and (ii) expectation of cure. These data are presented in Table 3

**Table 3:** Influence of type of interpretation, if any, on understanding of disease status and expectation of cure

	***N***	**% accuracy in understanding disease status^a^**	***N***	**Mean difference in % expectation of cure compared with doctors^b^**
Own language	20	60	25	26.2
Family interpreter	33	67	40	41.5
Other interpreter	5	60	7	28.6
Consultation in English without interpretation	32	44	39	41.0

a *P*=0.30.

b =0.26 for overall comparisons across groups.

. There are nonsignificant trends for patients who received their initial consultation in English (without interpretation) to have less accurate knowledge of disease status than those who had a consultation in their own language or for whom interpretation was provided. There were also trends for those with an initial consultation in English or with a family member interpreting to have the most unrealistic expectations of cure.

### Quality of life

Very few patients found the QoL questionnaire to be upsetting (0–3%) or indiscreet (0–5%), although some of the English-speaking patients found some questions to be annoying (15% compared to ⩽1% for the LEP patients).

**Figure 4:**
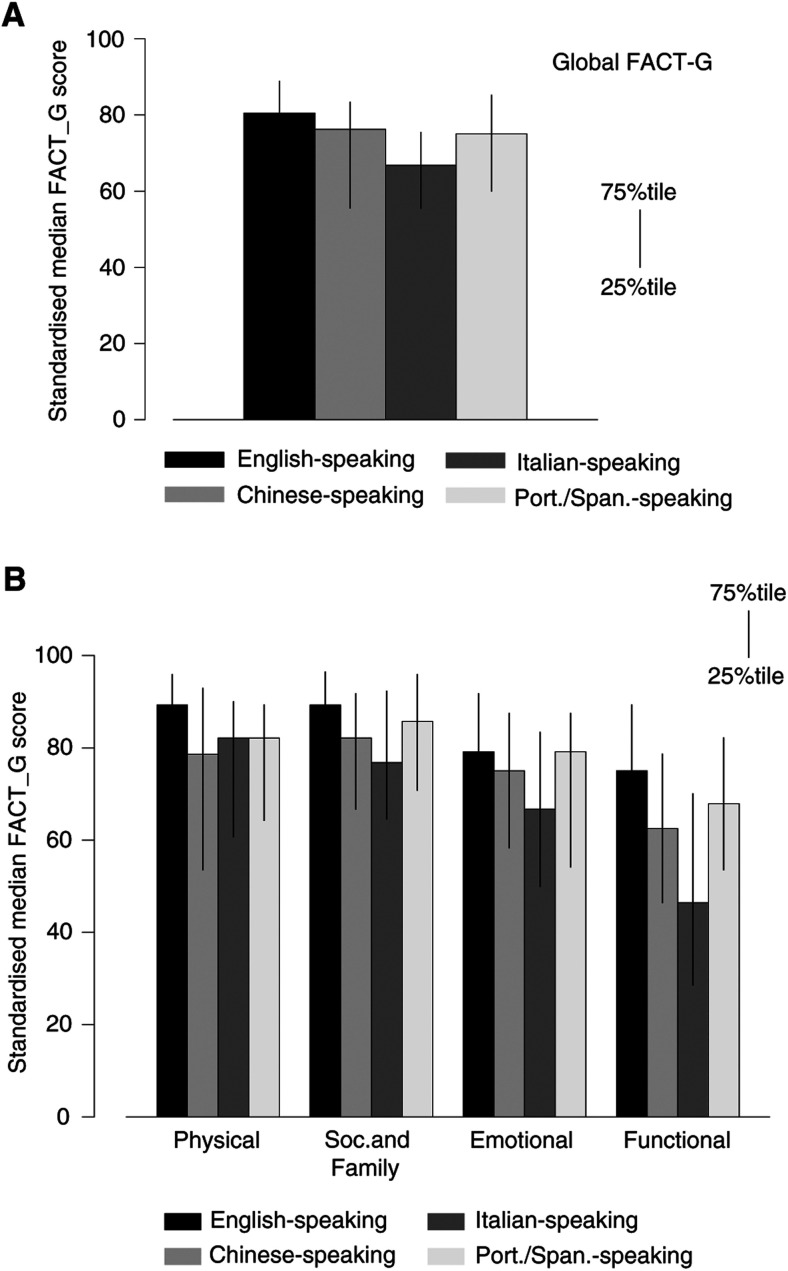
Effect of language group on (**A**) Overall Quality of Life Score, and (**B**) Different domains of Quality of Life.

The comparison of QoL among the ethnic groups is shown in Figure 4

, where higher scores indicate better QoL. The Italian-speaking patients had the poorest QoL, both in their global evaluation, and in the domains of Social-Family Well-being, Emotional Well-being and Functional Well-being. Differences between Italian-speaking patients and other groups were statistically significant (*P*=0.001) for global QoL and for Physical and Functional Well-being (*P*=0.03 and *P*=0.0001, respectively) in the crosscultural comparisons. Although more Italian-speaking patients were retired, removing the items in the FACT-G related to work had no effect on these differences in evaluation of QoL. The inclusion of other possible confounding variables in a multivariate analysis (see below) also did not change the conclusion that the Italian group had the poorest QoL. Each domain was rated on the questionnaire to be about equally important by each group, although absolute levels of the rating scales used were lower among Chinese-speaking patients (data not shown).

We have evaluated the interaction between QoL and language group with the inclusion of the possible confounding factors of status of disease (local, locoregional or metastatic), gender, age, marital status and level of education (Table 4

**Table 4:** (A) Influence of various factors on overall QoL (rated by the patient). (B) Influence of the same factors on Performance status (rated by the physician or research assistant)

	***P*-value^a^**	
**Factor**	**Univariate analysis**	**Multivariate analysis**
(A) Status of disease	0.017	0.0023
Ethnic group		
Chinese *vs* English	0.058	0.10
Italian *vs* English	0.0001	0.006
Spanish *vs* English	0.10	0.19
University education	0.0014	0.05
Age	0.067	0.66
Gender	0.32	0.17
Marital status	0.71	0.57
		
(B) Status of disease	0.008	0.002
Ethnic group		
Chinese *vs* English	0.048	0.04
Italian *vs* English	0.024	0.21
Spanish *vs* English	0.01	0.07
University education	0.0687	0.14
Age	0.3278	0.78
Gender	0.0689	0.18
Marital status	0.13	0.46

a Since *P*-values are not corrected for multiplicity, the level of significance is set at *P*<0.01.

). By univariate analysis, we found ethnic origin, status of disease and education to have a significant influence on QoL. In the multivariate analysis, status of disease predicted QoL (*P*=0.002), and being Italian-speaking predicted poorer QoL compared with English-speaking patients (*P*=0.006), with only nonsignificant trends when the other groups were compared to English-speakers. We undertook a similar analysis of the effects of the same factors on Performance status (assessed by the research assistant) which, as expected, was highly correlated (*P*<0.0001) with QoL. Not surprisingly, the major factor influencing Performance status was the status of disease (*P*=0.002), and there were only nonsignificant trends for those of non-English language groups to have poorer Performance status than English-speaking patients.

We also undertook supplementary analyses with (a) only socioeconomic and demographic variables in the model and (b) with language also included. The model without language can interpret about 9% of the variation in QoL, and language can account for about 3% more variation. After controlling for the sociodemographic factors, language was still a significant predictor of QoL.

There was no correlation between accuracy of knowledge of stage of disease (local, locoregional or metastatic) and QoL. There was, however, a correlation between the difference in estimates of cure by physician and patient, and overall QoL (*P*=0.005), with patients having more realistic estimates of probability of cure having better QoL. In the exploratory analysis with each domain of QoL, there was also correlation of accuracy of estimate of cure with functional and physical domains (*P*=0.002 and 0.0007, respectively), but not with emotional or social/family domains.

## DISCUSSION

This study provides support for the hypothesis that patients whose first language is English have better knowledge and understanding of their disease than those in the other language groups who were of Southern European, South American or Chinese origin. There is also some support for our second hypothesis, since although accurate knowledge of disease stage had no detectable effect on QoL, patients whose expectation of cure were closer to that of their physicians had better QoL.

Our study has several limitations. Our sample size of 202 patients was chosen for reasons of feasibility and while it was adequate to detect several important differences between the language groups, the study lacked power to evaluate other aspects such as language of consultation. All of the results will require confirmation in an independent study. The Italian-speaking group appeared to have particularly poor knowledge of disease and poor QoL. However, many more Italian-speaking patients declined to participate (for unknown reasons), and they also tended to be older and less educated (Table 1). Although being Italian-speaking predicted for poorer QoL in the multivariate analysis that included other possible confounding variables, these socioeconomic differences may have introduced bias that might explain, in part, our findings. Almost all of the LEP patients were first-generation immigrants to Canada and it is not possible to separate the effects of LEP and ethnic community within Canada from the influence of immigration. Most patients received consultation in English with either no translation, or translation provided by a family member, and we are unable to assess the benefits of consultation in the native language or of objective professional translators.

All groups had an inappropriately optimistic view of their chance of cure, in agreement with other studies (Daugherty *et al*, 1995; Cheng *et al*, 2000), and patients with LEP had poorer understanding of the status of their disease and their expectation of cure. This may be due to poor communication with the doctor, although cultural issues leading to denial or suppression of unwelcome information may play a role, as well as the desire to retain hope for cure (Mitchell, 1998). There are systematic variations across ethnic groups in experience with illness, expectations from treatment and acceptability of treatment (Juarez *et al*, 1999). Ethnic origin and social position are known to influence health-related behaviour, perceptions and meaning of life; they may also influence explanations about sickness by health professionals (Kleinman and Mendelsohn, 1978). There have been few studies of knowledge of disease status in cancer patients of different ethnic origins, but in general Latino and Black patients appear to have less accurate knowledge than Whites and Asians (Michielutte and Diseker, 1982; Stone and Siegel, 1986; Perez-Stable *et al*, 1992; Meyerowitz *et al*, 1998). We found that patients with LEP are less satisfied with information received and would like more information about their disease. This indicates a need for patient education and information to be provided in the patient's own language, or with professional interpreters who are less likely to modify information given by the physician than family members.

Our study suggests that Italian-speaking patients had poorest and English-speaking patients best QoL, although only differences between these two groups were statistically significant (Figure 4). Differences in the Physical, Emotional, Functional and Social/Family Well-being domains of QoL were of similar direction among the ethnic groups. It is unknown whether this represents true differences in QoL or a different frame of reference for interpreting QoL between cultures. Although not definitive, our findings are consistent with a greater effect of ethnic background on patient-evaluated QoL than on Performance status evaluated by an objective observer. A relationship between QoL and accuracy of knowledge about prognosis is also supported by our study.

Although QoL instruments are now being applied widely in international clinical trials that involve patients from diverse cultural backgrounds, only a few published studies have assessed the influence of ethnic origin on patient-rated QoL (Bernhard *et al*, 1998; de Haes and Olschewski, 1998). Equivalence has been achieved in the evaluation of QoL among different populations participating in some clinical trials but not in others (Hurny, 2000). In a study using the Rotterdam Symptom Checklist to assess aspects of QoL in a crosscultural trial of adjuvant chemotherapy and hormonal therapy for early breast cancer, there were significant differences in both baseline QoL, and changes in QoL across cultures (de Haes and Olschewski M). Significant differences in QoL between cultures before the initiation of adjuvant chemotherapy were also observed in studies by the International Breast Cancer Study Group (Hurny *et al*, 1992; Bernhard *et al*, 1998). A smaller study of the psychological adjustment of cancer patients with high performance status undergoing radiotherapy in Belgium and Turkey showed that Belgian patients experienced less psychological distress than Turkish patients before starting radiotherapy (Erbil *et al*, 1996). These findings are unlikely to be due only to translation of the questionnaires since rigorous methods have been used to develop QoL instruments in different languages (Aaronson *et al*, 1992; Ware *et al*, 1995,^1998^; Bonomi *et al*, 1996). The differences may be related to socioeconomic status, education, access to health care, language, knowledge, attitude toward doctors, treatment, patient behaviour and other confounding factors (Adler *et al*, 1994; National Coalition of Hispanic Health and Human Services Organizations, 1995). There is a limited understanding of how patients from different cultural backgrounds assess their QoL and what implications these cultural differences might have on measurement of QoL in the context of international clinical trials in cancer research. The differences among language groups disclosed by the present study and others suggest caution in generalising the effects of interventions on QoL from a study of one homogenous population to patients of different ethnic and cultural origins.
